# The Stop Codon after the nsp3 Gene of Ross River Virus (RRV) Is Not Essential for Virus Replication in Three Cell Lines Tested, but RRV Replication Is Attenuated in HEK 293T Cells

**DOI:** 10.3390/v16071033

**Published:** 2024-06-27

**Authors:** Christin Schmidt, Julia Gerbeth, Christine von Rhein, Florian D. Hastert, Barbara S. Schnierle

**Affiliations:** Section AIDS and Newly Emerging Pathogens, Department of Virology, Paul-Ehrlich-Institut, Paul-Ehrlich-Strasse 51-59, 63225 Langen, Germany

**Keywords:** Ross River virus, chikungunya virus, mCherry, non-structural proteins

## Abstract

A recombinant Ross River virus (RRV) that contains the fluorescent protein mCherry fused to the non-structural protein 3 (nsP3) was constructed, which allowed real-time imaging of viral replication. RRV-mCherry contained either the natural opal stop codon after the nsP3 gene or was constructed without a stop codon. The mCherry fusion protein did not interfere with the viral life cycle and deletion of the stop codon did not change the replication capacity of RRV-mCherry. Comparison of RRV-mCherry and chikungunya virus-mCherry infections, however, showed a cell type-dependent delay in RRV-mCherry replication in HEK 293T cells. This delay was not caused by differences in cell entry, but rather by an impeded nsP expression caused by the RRV inhibitor ZAP (zinc finger CCCH-Type, antiviral 1). The data indicate that viral replication of alphaviruses is cell-type dependent, and might be unique for each alphavirus.

## 1. Introduction

Alphaviruses are transmitted by mosquito vectors and cause a wide range of diseases in humans and animals. The Old World (OW) alphaviruses, like chikungunya virus (CHIKV), O’nyong-nyong virus (ONNV), Semliki Forest virus (SFV), Ross River virus (RRV), and Mayaro virus (MAYV), cause arthralgia. In contrast, viruses of the New World (NW) group, like Venezuelan (VEEV), Eastern (EEEV), and Western equine encephalitis virus (WEEV), cause encephalitis. The clinical signs of the acute diseases of OW alphaviruses are characterized by a febrile illness lasting 3–7 days, rash, and severe, sometimes long-lasting, arthralgia [1,2,3].

CHIKV is the most prevalent mosquito-borne alphavirus and infections are a public health challenge [2,4]. CHIKV was first described during an outbreak in southern Tanzania in 1952, but since then has been identified in over 100 countries [5,6]. CHIKV spread was rapidly accelerated through a single point mutation in the E1 protein, which allowed transmission by *Ae. albopictus* mosquitoes [7]. In contrast to the previous main vector *Ae. aegypti*, which is confined to the tropics and sub-tropics, *Ae. albopictus* also occurs in temperate and even cold temperate regions, and has spread from Asia to moderate climate zones in Africa, Europe, and the Americas [8]. The potential for CHIKV epidemics in naïve populations remains high [4].

RRV is endemic in Australia, Papua New Guinea, and islands of the South Pacific, and causes disease in humans and horses. Its geographic range was thought to be limited by the distribution of its primary reservoir hosts, initially believed to be marsupials; however, recent evidence suggests that RRV circulates endemically in the Pacific Islands, where marsupials are absent [9]. In addition, RRV is a vector generalist able to be transmitted by 40 species of mosquito, which poses a serious risk of wider dissemination [10]. In contrast to CHIKV, about 70% of RRV infections are asymptomatic (compared to 28% of CHIKV infections) [11,12]. RRV disease is non-lethal and self-limiting, but CHIKV causes a low rate of mortality (0.13%) [13]. About 50% of RRV- and CHIKV-infected individuals will develop chronic disease with debilitating arthralgia [14,15]. Consequently, RRV is an important public health concern in Australia with economic costs of about USD 4.3 million per year [16,17].

**Figure 1:**
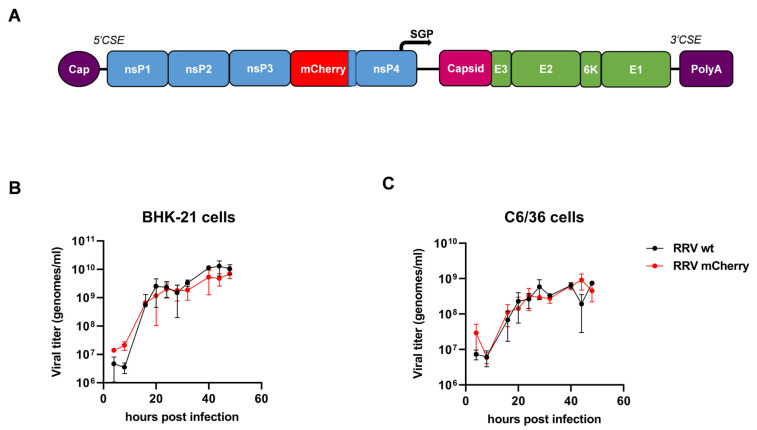
RRV-mCherry genome and replication. (**A**) The mCherry gene was inserted after nsP3 in front of the opal stop codon. CSE: cis-acting or conserved sequence elements; SGP: subgenomic promotor. (**B**) BHK-21 and (**C**) C6/36 cells were infected with wt RRV-mCherry or RRV wt at an MOI of 1. Cell supernatants were collected at 4, 8, 16, 20, 24, 28, 32, 40, 44, and 48 hpi and viral RNA was quantified by RT-qPCR. Viral titers were determined by comparison to an internal standard and are indicated as genomes/mL. The mean values ± standard deviations of two independent experiments are indicated.

Alphaviruses have a single-stranded, positive-sensed RNA genome of approximately 12 kb that contains two open-reading frames encoding either the four non-structural (nsP1–4) or the structural proteins (capsid and the envelope proteins E3-E2-6K-E1; see Figure 1A). Upon infection, and release of the genomic RNA (gRNA) into the cytoplasm, the nsPs are directly translated into a non-structural precursor protein. The nsPs have enzymatic activities and accomplish viral RNA replication. Early in infection, the nonstructural proteins consist of the polyprotein precursor nsP123 and nsP4. A conserved opal stop codon at the C terminus of the nsP3 gene causes occasionally readthrough during translation in several alphaviruses and produces full-length nsP1234 [18]. Therefore, nsP4 concentrations are much lower than that of nsP1-3 [18]. The nsP1234 protein is cleaved by the nsP2 protease, releasing nsP4, the RNA-dependent RNA polymerase. The polyprotein nsP123 and cleaved nsP4 mediate the synthesis of the minus-sense RNA which serves as a template for the gRNA and the subgenomic RNA (sgRNA), which is synthesized under the control of the subgenomic promoter (sgP). The structural proteins are then translated from the sgRNA [18].

Like most alphaviruses, RRV and CHIKV encode a opal stop codon after the nsP3 gene, however, some CHIKV isolates encode an arginine residue in place of the opal termination codon [19]. For CHIKV, this mutation did not impair viral replication kinetics in vitro or in vivo, but showed an attenuation of arthritis and pathology in a mouse model [20]. ONNV, an alphavirus closely related to CHIKV, encodes either an opal termination codon or an arginine codon and the presence of the opal codon provided a fitness advantage in mosquito cells [21].

The relevance of the opal stop codon behind the RRV nsp3 gene has not been studied before. Here, we generated an RRV encoding an nsP3-mCherry fusion protein and a variant that has the opal stop codon deleted. The replication of the two viruses was analyzed in different cell lines. Using these viruses, no significant effect of the opal stop codon on viral replication was observed, but cell-specific differences in RRV replication compared to CHIKV were detected.

## 2. Materials and Methods

### 2.1. Cell Culture

All mammalian cells used were cultured at 37 °C under 5% CO_2_. HEK 293T (CRL-11268), HEK 293T ZAP KO [22] (a generous gift of Paul Bieniasz, Rockefeller University, New York, USA and Frank Kirchhoff, University Ulm, Germany) and Vero E6 (CRL-1586) cells were grown in Dulbecco’s modified Eagle medium (DMEM; Lonza, Verviers, Belgium). BHK-21 (CCL-10) cells were incubated in Roswell Park Memorial Institute medium (RPMI; Biowest, Nuaille, France). All media were supplemented with 10% fetal bovine serum (FBS) (PAA, Pasching, Austria), 1% L-glutamine (200 mM; Lonza, Verviers, Belgium), and 1% penicillin-streptomycin (Fisher Scientific, Schwerte, Germany). The *Aedes albopictus* C6/36 (CRL-1660) cells were grow at 28 °C without any extra CO_2_ or humidity in Leibovitz L15 medium (ThermoFisher, Dreieich, Germany) supplemented with 10% FBS Gold (ThermoFisher, Dreieich, Germany), 1% non-essential amino acids (ThermoFisher, Dreieich, Germany), 1% L-glutamine, and 1% penicillin-streptomycin.

### 2.2. Generation and Production of RRV-mCherry and CHIKV-mCherry

The *nsP3-mCherry* fusion gene was generated by replacing an Nsi I and Xba I fragment from the RRV cDNA clone pRR64 (kind gift from Richard Kuhn [23]) with a synthetic gene containing the RRV flanking sequences and the *mCherry* gene. The *mCherry* sequences were inserted after the *nsP3* sequence, directly in front of the opal stop codon. The variant without the stop codon was also replaced as an Nsi I and Xba I fragment using a synthetic DNA fragment.

To produce RRV-mCherry RNA, the plasmid was linearized by Sac I restriction digestion. Using the SP6 promoter included in the plasmid, single-stranded RNA was in vitro transcribed using the HiScribe SP6 RNA Synthesis Kit (according to the manufacturer’s protocol; NEB, Frankfurt/Main, Germany). BHK-21 cells in 10 cm dishes were transfected with 10 µg of RNA to produce RRV-mCherry and the virus was subsequently amplified on BHK-21 cells. After being concentrated 100-fold by ultracentrifugation (280,000 rpm, 2 h, 4 °C), the virus stock was aliquoted and stored at −80 °C. The titer was determined by plaque assay from frozen aliquots by titration on Vero E6 cells and is given as plaque forming units (pfu) per ml. CHIKV-mCherry was produced as described previously [24,25]. For comparison of wt RRV-mCherry and del RRV-mCherry virus supernatants were used without concentration by ultracentrifugation.

### 2.3. Qualitative Reverse Transcription (RT)-PCR Analysis

Viral RNA as a template was amplified using the OneTaq^®^ One-Step RT-PCR Kit (NEB, Frankfurt/Main, Germany). In short, 100 ng template RNA was mixed with 400 nM specific primers (RRV-mCherry fw: 5′-GCCTCGGTATGCAGTGTGA; RRV-mCherry rev: 5′-CCTATCTATGATCACTGCCTTCATGT), 25 μL reaction mix, and 2 μL enzyme mix in 50 μL RNase-free water to synthesize cDNA. The PCR cycles were carried out using a thermocycler. The amplified DNA fragments were separated by agarose gel electrophoresis for analysis and were subsequently sequenced (Eurofins Genomics, Ebersberg, Germany).

### 2.4. RT-Quantitative PCR (RT-qPCR)

A one-step RT-qPCR to quantify viral RNA was performed with the LightCycler Multiplex RNA Virus Master (Roche, Mannheim, Germany) following the manufacturer’s instructions. In short, for each reaction, 5 µL RNA extracted from cell supernatants were mixed with 4 µL RT-PCR Reaction Mix 5×, 8 pmol of each specific forward and reverse primer (RRV qPCR fw: 5′-CCGTGGCGGGTATTATCAAT; RRV qPCR rev: 5′-AACACTCCCGTCGACAACAGA), 4 pmol of the corresponding hydrolysis probe (RRV qPCR probe: 5′-ATTAAGAGTGTAGCCATCC), and 0.1 µL RT Enzyme Solution, adding RNase-free water to 20 µL. An internal standard, prepared from a virus stock of known titer, was treated the same as the samples. RT-qPCR was performed with the BioRad Thermal Cycler CFX96 Real Time System (BioRad, Munich, Germany). The standard PCR cycle program was applied, according to the manufacturer’s manual. The Software BioRad CFX Manager (Version 3.1) was used to analyze the results. A standard curve was automatically generated, which was used to calculate the amount of virus genomes from cycle threshold (Ct).

### 2.5. Virus Infection Kinetics by Flow Cytometry

To evaluate the infection kinetics of RRV- and CHIKV-mCherry, cells were infected at a multiplicity of infection (MOI) as indicated. The cells were fixed with 2% paraformaldehyde (PFA) in PBS at the indicated time points. Analysis was performed using a BD LSRFortessa cytometer and the software FACSDiva (V9) (BD, Heidelberg, Germany).

Virus replication after viral genomic RNA transfection was performed with cells seeded in 12-well plates and transfected with 1 µg of CHIKV-mCherry or RRV-mCherry RNA using Lipofectamine™ MessengerMAX™ (according to the manufacturer’s protocol; ThermoFisher, Darmstadt, Germany). The cells were fixed with 2% PFA in PBS at the indicated time points and analyzed using a BD LSRFortessa cytometer.

### 2.6. RNA Isolation

The Direct-zol™ RNA MiniPrep Kit (Zymo Research, Freiburg, Germany) was used to purify total RNA from supernatants or cell lysates. In accordance with the manufacturer’s manual, samples were inactivated, bound to a spin column, and washed before the RNA was eluted.

### 2.7. Immunostaining of Cells

To determine the location of nsP3-mCherry and the GTPase-activating protein (SH3 domain)-binding proteins 1 (G3BP-1) during CHIKV- or RRV-mCherry infections, HEK 293T and BHK-21 cells were infected at an MOI of 1 with CHIKV- or RRV-mCherry. After 18 h, cells were fixed with 2% PFA in PBS and permeabilized with 0.5% Triton X-100 (Biozol, Leipzig, Germany) in PBS. Cells were subsequently immunostained using an antibody against G3BP (Aviva Systems Biology, San Diego, CA, USA) followed by an anti-rabbit FITC-conjugated secondary antibody, and counterstained with 4′, 6-diamidino-2-phenylindole (DAPI) (1 μg/mL; Sigma-Aldrich, Taufkirchen, Germany). Samples were imaged with a Leica Stellaris 8 microscope equipped with a 93× objective (HC PL APO 40×/1.10 W CORR CS2).

### 2.8. Western Blot Analysis

Protein expression was evaluated from cell lysates by Western blot analysis. Cell lysates were prepared and the protein concentrations were determined with the Pierce™ BCA™ Protein-Assay Kit (ThermoFisher Scientific, Schwerte, Germany). For Western blot analyses, 20 µg protein were loaded per slot onto a gel and separated by SDS-PAGE. Proteins were transferred onto polyvinylidene difluoride (PVDF) membranes with a Bio-Rad semidry blotter. Membranes were blocked with Roti-Block (Carl Roth, Karlsruhe, Germany) and proteins were then detected with primary antibodies directed against mCherry (Abcam, Cambridge, UK, # ab183628), huZAP (Abcam, Cambridge, UK, # ab154680) and β-actin (Sigma, Munich, Germany; #A5441), and appropriate secondary HRP-coupled antibodies. Detection was performed with the ECL detection system (Amersham, Freiburg, Germany) and Fusion FX7 (Vilber, Eberhardzell, Germany).

### 2.9. Lentiviral Vector Particle Production

Lentiviral vectors were produced as described before [26]. HEK 293T cells in 10 cm dishes were cotransfected using Lipofectamine^®^ 2000 (according to the manufacturer’s protocol; ThermoFisher, Darmstadt, Germany) with the following amounts of plasmid DNA: 10 µg pCSII-Luc, 6.5 µg pMDLg/pRRE, 2.5 µg pRSVrev, 5.3 µg pIRES2-eGFP-CHIKV or pcDNA-RRV. After 24 h of incubation, the medium was replaced with 5 mL of fresh DMEM per dish. Twenty-four hours later, the supernatant containing vector particles was harvested, sterile filtered with 0.45 µm filters (Sartorius, Göttingen, Germany), and ultracentrifuged (1 h at 50,000 rpm, rotor TLA 100.3; Optima TLX Ultracentrifuge, Beckman Coulter, Krefeld, Germany) to concentrate the volume 100-fold; the particles were then resuspended in DMEM and frozen in aliquots at −80 °C.

### 2.10. Titration of Lentiviral Vector Particles

The titer of the luciferase-encoding lentiviral vectors was determined by transduction of cells with serial two-fold dilutions of vector particles in triplicates. For this, 6000 cells in 20 µL DMEM (supplemented with 1% FBS) were seeded per well into white CELLSTAR 384-well microtiter plates (Greiner Bio-One, Frickenhausen, Germany). After 20 h of incubation at 37 °C, cell transduction was determined by addition of 10 μL BriteLite (PerkinElmer, Rodgau, Germany) substrate to each well. The resulting luminescent luciferase signal was detected as counts/s with the Tecan Spark reader (Tecan, Männedorf, Switzerland) for 1 s per well.

### 2.11. Statistical Analysis

Mean values and standard deviations were calculated with Excel. For statistical analysis, *t*-tests and one-way or two-way analyses of variance (ANOVA) with appropriate post-tests, as indicated in the figure legends, were performed using GraphPad Prism 7.04 (GraphPad Software, La Jolla, CA, USA).

## 3. Results

### 3.1. Generation and Characterization of a Recombinant RRV-mCherry

The novel recombinant virus, RRV-mCherry, was generated by insertion of the *mCherry* gene into the RRV genome between the *nsP3* and *nsP4* genes [23]. Thereby, an *nsP3-mCherry* fusion gene was generated (Figure 1A). A similar design was used by Kümmerer et al. to construct a recombinant CHIKV-mCherry [24]. In vitro-transcribed single-stranded RNA was used to produce RRV-mCherry in BHK-21 cells. The recombinant virus was first characterized. We determined the stability of the gene insertion during serial passages of the virus in BHK-21 cells. The cells were infected with RRV-mCherry at an MOI of 0.01. After 24 h, the cell supernatant was used to infect new BHK-21 cells. This procedure was repeated four times. RNA was subsequently extracted from the supernatants and RT-PCR was performed, amplifying the DNA surrounding the *mCherry* gene. Sequencing of the PCR fragments revealed no alterations in the sequences and confirmed the stability of the mCherry insertion.

To further ensure that the in vitro replication of RRV-mCherry was not altered, the growth kinetics were compared to wild-type RRV (RRV wt) generated from the original RRV cDNA clone pRR64 [23]. The growth characteristics were compared in BHK-21 cells and the *Ae. albopictus* cell line C6/36. The cells were infected with RRV-mCherry or RRV wt at an MOI of 1 and the cell supernatants were collected at several time points after infection. The amount of virus was determined by RT-qPCR and normalized to a virus stock of known titer; values are indicated as genomes per ml. No difference in the growth kinetics were observed between RRV-mCherry and RRV wt, neither in mammalian cells nor in insect cells (Figure 1B,C). However, about 1 log more virus was produced in BHK-21 cells than in C6/36 cells.

**Figure 2:**
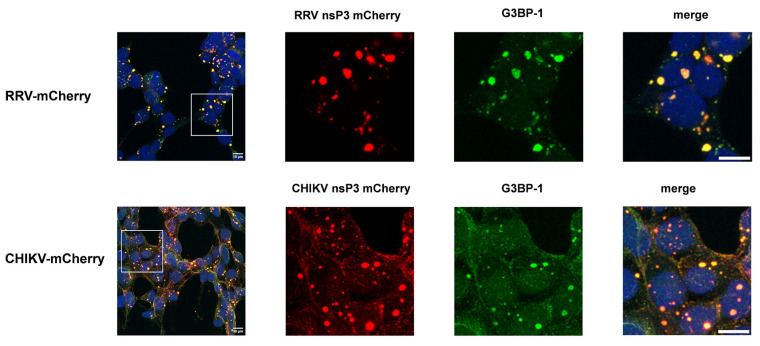
Assembly of nsP3-containing complexes. HEK 293T cells were infected with RRV- and CHIKV-mCherry at an MOI of 1 and fixed after 18 h. The cell nuclei were stained with DAPI (blue) and G3BP-1 with a specific antibody (green); nsP3 is visible due to the red mCherry fluorescence. Representative pictures of two independent experiments are shown. The images were taken at 93× magnification and the scale bars in the merge images correspond to 10 µm.

The characterization of RRV-mCherry demonstrated a stable insertion of the reporter gene that did not alter the growth kinetics. In addition, localization of the nsP3-mCherry fusion protein was analyzed in RRV-mCherry infected HEK 293T cells. NsP3-mCherry localized in dot-shaped accumulations in the cell cytoplasm (Figure 2). Staining of the cells with an antibody directed against G3BP-1 showed the well-documented colocalization of nsP3-mCherry and the G3PB-1 protein (Figure 2) [27]. The same staining was obtained with CHIKV-mCherry infected HEK 293T (Figure 2 lower panel). Accordingly, this virus was suitable for studying RRV replication.

### 3.2. Comparison of wt RRV-mCherry and del RRV-mCherry

**Figure 3:**
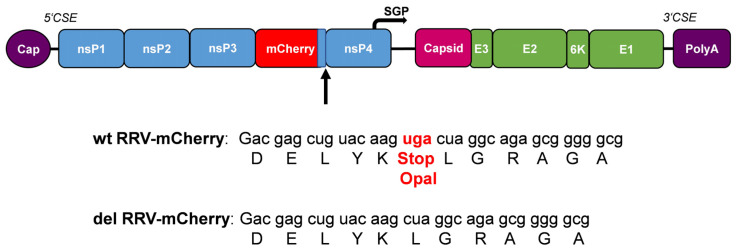
RRV-mCherry genome and opal stop codon. The location of the stop codon after the nsP3 gene is indicated. Wt RRV-mCherry contains the opal stop codon and del RRV-mCherry has the stop codon deleted.

To evaluate the effect of the opal stop codon, a recombinant RRV genome was similarly constructed by using a synthetic DNA fragment containing the mCherry gene flanked by nsP3 and nsP4 sequences without the opal stop codon after the nsP3 gene (del RRV-mCherry) (Figure 3).

**Figure 4:**
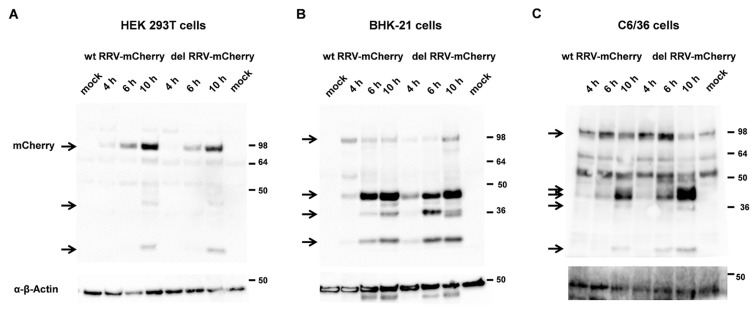
Western blot analysis of nsP3-mCherry expression. (**A**) HEK 293T, (**B**) BHK-21, or (**C**) C6/36 cells were infected at an MOI of 1 and cells were harvested at 4, 6, and 10 hpi. Cell lysates were separated by SDS/PAGE and the nsp3-mCherry protein was detected with an antibody directed against the mCherry protein. This antibody cross-reacts with a protein sightly larger than nsP3-mCherry and lower migrating bands in C6/36 cell lysates. NsP3-mCherry and its degradation products are indicated by an arrow. Loading was analyzed with an antibody directed against β-actin. The expected molecular weight of nsP3-mCherry is 88 kDa and 42 kDa for β-actin.

The del RRV-mCherry variant should synthesize a nsP1-4 protein with stoichiometric amounts of nsP3 and nsP4. Wt RRV-mCherry and del RRV-mCherry were produced from in vitro-transcribed single-stranded RNA by transfection into BHK-21 cells. The experiments were performed with virus after three passages on BHK-21 cells. The titer was determined by plaque assay and titration on Vero E6 cells and the virus genomes were analyzed by RT-PCR and sequencing. The deletion of the stop codon and the wt sequences were preserved correspondingly indicating that del RRV-mCherry was stable during virus amplification. We first aimed to compare the infection kinetics of the two RRV-mCherry viruses in the cell lines HEK 293T, BHK-21 and C6/36 insect cells. The cells were infected at a MOI of 1, and first analyzed by Western blot analysis of the nsP3-mCherry fusion protein (Figure 4). Although the deletion of the stop codon should generate an nsp1-4 fusion protein, there was no difference detectable in the molecular weight of the nsP3-mCherry protein from wt RRV or del RRV-mCherry, even at the early time point of 2 h after infection (hpi). At later time points nsP3-mCherry started to become degraded and lower migrating bands appeared (Figure 4A). Compared to HEK 293T cells, BHK-21 cells were much faster infected and after 4 hpi full length nsP3-mCherry was hardly detectable (Figure 4B). In C6/36 insect cells, these degradation products appeared at 10 hpi (Figure 4C). However, there were no substantial difference in the nsP3-mCherry amounts between wt RRV- or del RRV-mCherry-infected cells.

**Figure 5:**
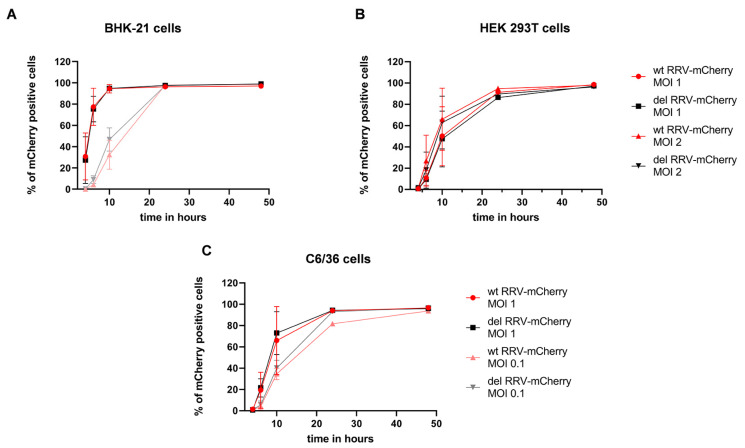
Virus replication kinetics in cell lines. (**A**) BHK-21, (**B**) HEK 293T, or (**C**) C6/36 cells were infected at the indicated MOIs. The percentage of mCherry-positive cells was determined by flow cytometry at 4, 6, 10, 24, and 48 h. The mean values ± standard deviations from at least three independent experiments are indicated. For statistical analysis, an unpaired *t*-test was performed and showed no significant differences between wt RRV- and del RRV-mCherry infections.

Additionally, the cells were infected at different MOIs and the percentage of mCherry-positive cells was determined by flow cytometry after 4, 6, 10, 24, and 48 h. Again, no significant differences in the infection kinetics of the two viruses were observed in all three cell lines (Figure 5). Accordingly, the presence of the opal stop codon had no influence on RRV replication kinetics in the studied mammalian or insect cell lines.

### 3.3. RRV-Mcherry Replication Is Delayed in HEK 239T Cells

**Figure 6:**
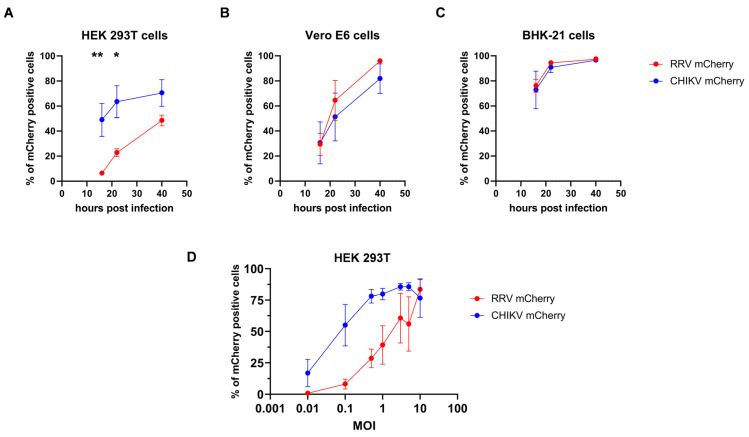
Comparison of RRV-mCherry and CHIKV-mCherry replication kinetics. The replication kinetics of RRV-mCherry and CHIKV-mCherry were determined in (**A**) HEK 293T, (**B**) Vero E6, and (**C**) BHK-21 cells. Cells were infected at an MOI of 0.1 and the percentage of infected cells was determined by flow cytometric analysis. The mean values ± standard deviations of five repeats are plotted. RRV-mCherry is depicted in red and CHIKV-mCherry in blue. (**D**) HEK 293T cells were infected at the indicated MOIs and analyzed 16 h after infection. For statistical analysis, a two-way ANOVA followed by Sidak’s multiple comparison was performed (* *p* < 0.05; ** *p* < 0.01).

Yet, a clear cell-specific difference in RRV infection became apparent from the above experiments (Figure 5). When HEK 293T cells were infected at an MOI of 1, 6 hpi, about 12% of the cells were mCherry positive (Figure 5B). Infected BHK-21 cells exhibited at 6 hpi 85% mCherry-positive cells (Figure 5A). Increasing the MOI to 2 for the HEK 293T cell infection also showed a delay in RRV replication compared to BHK-21 at an MOI of 1 (Figure 5A,B). We therefore asked if this a general behavior of alphaviruses or if it is specific for RRV. RRV-mCherry allowed a direct comparison of RRV replication with CHIKV-mCherry [24]. Consequently, we compared the infection kinetics of wt RRV-mCherry and CHIKV-mCherry in the cell lines HEK 293T, Vero E6, and BHK-21. The cells were infected at an MOI of 0.1 and the percentage of mCherry-positive, infected cells was determined by flow cytometry. Vero E6 and BHK-21 cells were efficiently infected by both viruses and either 50% of Vero E6 or 90% of BHK-21 cells were mCherry positive 24 h after infection (Figure 6B,C) and no significant differences in the infection kinetics were observed. After 48 h, almost all cells were infected by RRV-mCherry or CHIKV-mCherry. In contrast, HEK 293T cells were significantly less efficiently infected by RRV-mCherry (Figure 6A). The difference in infection was greatest at the earlier time points, and at 16 h after infection, only 3.7% of HEK 293T cells were infected by RRV-mCherry in comparison to 45% by CHIKV-mCherry (*p*-value of 0.0075). This difference diminished when higher MOIs were used for infection (Figure 6D). At an MOI of 10 no difference in infection was observed 16 h after infection.

**Figure 7:**
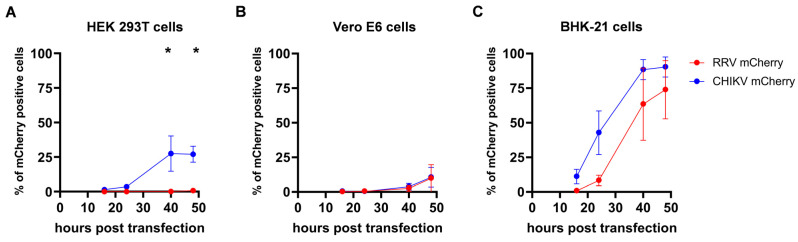
Virus replication after transfection of genomic RNA. (**A**) HEK 293T, (**B**) Vero E6, and (**C**) BHK-21 cells were transfected with 1 μg genomic RRV- or CHIKV-mCherry RNA and the percentage of mCherry-positive cells was determined by flow cytometry at the indicated time points. The mean values ± standard deviations from two independent experiments are indicated. For statistical analysis, a two-way ANOVA followed by Sidak’s multiple comparison was performed (* *p* < 0.05).

To assess if the differences between RRV and CHIKV infection of HEK 293T are caused by impaired cell entry, viral entry was circumvented by transfection of RRV- and CHIKV-mCherry genomic RNA. HEK 293T, Vero E6, or BHK-21 cells were transfected and viral replication was monitored as the number of mCherry-positive cells. In HEK 293T cells, CHIKV replication was significantly faster and RRV replication was massively delayed (Figure 7A). After 48 h, only 0.7% of cells expressed the RRV nsP3-mCherry fusion protein. In contrast, both viruses showed similar kinetics in Vero E6 cells (Figure 7B). In BHK-21 cells, CHIKV replication was slightly faster than RRV replication, and reached high values 40 h after transfection; however, this difference was not significant (Figure 7C). The data point to a delay in RRV non-structural protein synthesis and a reduced viral replication in HEK 293T cells, which are not caused by entry defects.

**Figure 8:**
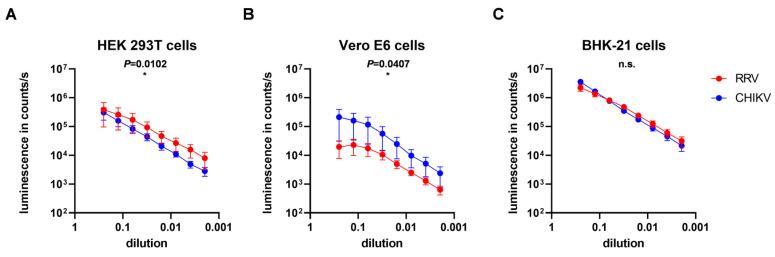
Transduction of cells with pseudotyped vector particles. (**A**) HEK 293T, (**B**) Vero E6, and (**C**) BHK-21 cells were transduced with lentiviral vectors carrying the respective envelope proteins. Transduction was determined as luminescence in counts per second. The mean values ± standard deviations of these relative luminescence obtained from three experiments carried out in triplicate are indicated. For statistical analysis, a paired two-tailed *t*-test was performed (* *p* < 0.05).

To further support that the difference between RRV and CHIKV infection of HEK 293T are caused by impaired cell entry, transduction by pseudotyped lentiviral vector particles encoding the luciferase gene was evaluated. As receptor binding and membrane fusion are carried out by the alphaviral glycoproteins, lentiviral vectors carrying either the RRV or the CHIKV envelope proteins thereby obtain the host range of the respective virus. Vectors containing the RRV or CHIKV glycoproteins E3–E1 and a luciferase reporter gene were used in two-fold dilutions to transduce the target cells [26]. Transduction, and thus cell entry, was quantified as luciferase units. HEK 293T cells showed a significantly higher level of transduction by RRV- than by CHIKV-pseudotyped vectors, indicating that RRV cell entry is efficient in HEK 293T cells and does not cause the impaired replication (Figure 8A). In contrast, Vero E6 cells showed slightly higher transduction by CHIKV-pseudotyped vectors (Figure 8B). Both vectors showed a 10-fold enhanced transduction of BHK-21 (Figure 8C), which correlates with the fast replication of the viruses in BHK-21 cells (Figure 6C).

**Figure 9:**
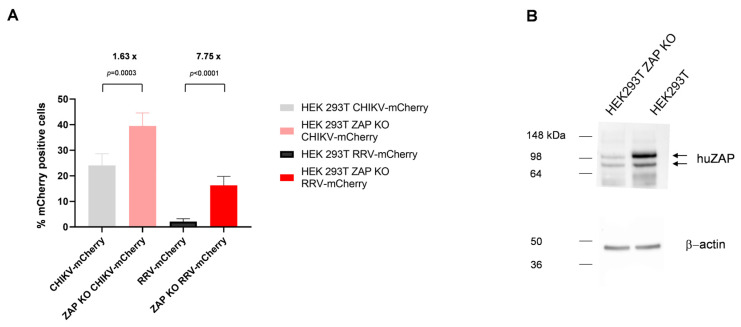
Virus replication in huZAP knock-out cells. HEK 293T ZAP KO cells were infected either with CHIKV- or RRV-mCherry at an MOI of 0.1. (**A**) The percentage of mCherry-positive cells in HEK 293T ZAP KO or HEK293T cells was determined at 10 hpi. The ratio of mCherry-positive cells in HEK 293T ZAP KO compared to HEK293T is given and the difference was highly significant as indicated by the *p* values. For statistical analysis, an unpaired *t*-test was performed. (**B**) HuZAP and actin expression was analyzed by Western blot analysis from cell lysates of HEK 293T ZAP KO or HEK293T cells using an huZAP- and β-actin-specific antibody. Actin was detected as loading control.

A cell-specific defect in RRV replication has been described before [28]. The authors showed that alphaviruses have differential sensitivity to the human antiviral effector molecule, zinc finger CCCH-type, antiviral protein 1 (huZAP) [29]. Therefore, we used HEK 293T ZAP KO cells, which are huZAP-deficient [22]. HEK 293T ZAP KO as well as HEK 293T cells were infected with CHIKV- or RRV-mCherry at an MOI of 0.1 and the cells were analyzed after 10 h. As indicated in Figure 9A, a 7.7-fold increase in RRV-mCherry infected cells compared to HEK 293T was detected in HEK 293T ZAP KO cells. In contrast, CHIKV-mCherry infection increased only 1.6-fold in HEK 293T ZAP KO cells. Although the ZAP knock-out was performed by CRISPR, residual ZAP protein was detected by Western blot analysis indicating that still some ZAP expressing cells were present (Figure 9B). However, even with this partial knock-down of ZAP, RRV replication was significantly increased. This indicates that the delayed replication of RRV in HEK 293T cells is very likely caused by huZAP.

## 4. Discussion

Fluorescent imaging is a versatile and sensitive tool that is based on detection of light emission from fluorescent molecules and proteins and was used here to study alphavirus replication. First, an RRV containing an nsP3-mCherry fusion protein was generated. As shown before for CHIKV-mCherry, this insertion does not interfere with RRV replication [24]. We used this virus to analyze the impact of the opal stop codon between nsP3 and nsP4.

For RRV and most other alphaviruses, the opal stop codon is six codons before the cleavage site between nsP3 and nsP4 and translational readthrough occurs at a low frequency and is assumed to regulate nsP4 levels [30]. However, some alphaviruses like the Sindbis virus strain AR86 and the SFV strain SFV4 carry a sense codon, which has been shown to be an important contributor to neurovirulence in adult mice [31,32]. Others reported for ONNV a fitness advantage in mosquito cells but not in mammalian cells by the presence of an opal codon between nsP3 and nsP4 [21]. Furthermore, changing the natural opal stop codon into an arginine codon in CHIKV was reported to decrease viral replication in C6/36 cells [33]. Others demonstrated recently that CHIKV replication either in mammalian or mosquito cells is not dependent on the presence or absence of an opal stop codon near the end of nsP3 [34]. Therefore, we were interested, if deletion of the opal stop codon in del RRV-mCherry would influence RRV-mCherry replication.

Our data show that deleting the opal stop codon had no effect on RRV replication in human, hamster, or mosquito cell lines. Furthermore, an nsP1-4 protein was not detectable by Western blot analysis and indicates that nsP4 is rapidly cleaved off the fusion protein by the viral protease. This suggests that the presence of stoichiometric amounts of nsP1-3 and nsP4 have no impact on RRV replication. It has been described before that the viral RNA polymerase, nsP4, is short-lived in infected cells and was found to be tightly regulated in infected cells and excess nsP4 was rapidly degraded by the N-end rule pathway [35]. Most likely, due to the short half-life of the nsP4 protein, surplus amounts of nsP4 do not influence RRV replication.

During our studies a clear cell-type difference in RRV replication became apparent and we questioned if this is restricted to RRV or also observed for other viruses like CHIKV. Therefore, CHIKV- and RRV-mCherry replication was analyzed in different cell lines. Both viruses, CHIKV-mCherry and RRV-mCherry, replicated with similar kinetics in Vero E6 and especially in BHK-21 cells, but RRV-mCherry replication was severely delayed in HEK 293T cells. The delayed onset of RRV-mCherry replication in HEK 293T cells was also observed when virus genomic RNA was transfected into cells and shows that it is not caused by differences in cell entry of the two viruses. However, recently it was reported that alphaviruses have differential sensitivity to the antiviral effector molecule, zinc finger antiviral protein (ZAP) [28,29]. RRV and Sindbis virus (SINV) were more sensitive to endogenous ZAP than ONNV and CHIKV in HEK 293T cells. In BHK-21 cells type I interferon stimulation has been described to be necessary for ZAP activity, but in HEK 293T cells ZAP is already functional [36,37]. ZAP has been described to bind viral RNA and support their degradation [38]. For SINV it has been shown before that ZAP blocks translation of incoming viral RNA by binding to its RNA [28]. Here, a reduction in huZAP expression in HEK 293T cells increased RRV replication. In contrast, CHIKV replication was only minimally altered (Figure 9). The ZAP KO cells were a bulk culture and still contain some ZAP expressing cells, which was observed by Western blot analysis in Figure 9B. These residual ZAP expressing cells interfere with RRV replication and therefore RRV replication did not reach the values obtained by CHIKV infection. This indicates that the effects observed by us make it very likely to be caused by ZAP hindering the translation of nsPs and thereby causing the delay of RRV replication in HEK 293T cells. However, when the MOI was increased to 10, no difference in the percentage of nsP3-mCherry-positive HEK 293T cells was detected between RRV and CHIKV (Figure 6D), indicating that the factor causing this impairment of RRV replication is present in limiting amounts. Our data support the central role of ZAP as a cellular defense factor against virus infections.

## 5. Conclusions

In summary, we observed that RRV replication is unaltered in vitro when the opal stop codon after nsP3 is removed. However, alphavirus replication varies in different in vitro cell culture models. RRV replication in human HEK 293T cells is most likely hindered by huZAP interfering with nsP translation. To characterize alphaviruses, multiple cell lines should be analyzed and viruses labeled with fluorescent proteins are perfect tools for this.

## Data Availability

The data presented in this study are available in the article.
